# The impact of Sangju Qingjie Decoction on the pulmonary microbiota in the prevention and treatment of chronic obstructive pulmonary disease

**DOI:** 10.3389/fcimb.2024.1379831

**Published:** 2024-04-30

**Authors:** Zheng Liu, Ying Huang, Chao Hu, Xiang Liu

**Affiliations:** ^1^ Clinical Pharmacy, Xiangtan Center Hospital, Xiangtan, Hunan, China; ^2^ Pulmonary and Critial Care Medicine, Zhongshan Hospital of Traditional Chinese Medicine, Zhongshan, Guangdong, China; ^3^ Pulmonary and Critial Care Medicine, Xiangtan Center Hospital, Xiangtan, Hunan, China

**Keywords:** SJQJD, COPD, pulmonary microbiota, biomarker, machine learning

## Abstract

**Objective:**

Exploring the effect of SJQJD on the pulmonary microbiota of chronic obstructive pulmonary disease (COPD) rats through 16S ribosomal RNA (rRNA) sequencing.

**Methods:**

A COPD rat model was constructed through smoking and lipopolysaccharide (LPS) stimulation, and the efficacy of SJQJD was evaluated by hematoxylin and eosin (H&E) staining and Enzyme-Linked Immunosorbnent Assay (ELISA). The alveolar lavage fluid of rats was subjected to 16S rRNA sequencing. The diversity of lung microbiota composition and community structure was analyzed and differential microbiota were screened. Additionally, machine learning algorithms were used for screening biomarkers of each group of the microbiota.

**Results:**

SJQJD could improve lung structure and inflammatory response in COPD rats. 16s rRNA sequencing analysis showed that SJQJD could significantly improve the abundance and diversity of bacterial communities in COPD rats. Through differential analysis and machine learning methods, potential microbial biomarkers were identified as *Mycoplasmataceae*, *Bacillaceae*, and *Lachnospiraceae*.

**Conclusion:**

SJQJD could improve tissue morphology and local inflammatory response in COPD rats, and its effect may be related to improve pulmonary microbiota.

## 1 Introduction

COPD is a common respiratory disease in clinical practice, and its risk factors include advancing age, long-term smoke irritation, high incidence, and mortality, with continuous airflow restriction being the main pathological characteristic. Relevant statistics (Fang et al., 2018) show that there are approximately 100 million patients with COPD and increasing incidences of COPD in China. According to the World Health Organization, COPD may become the third leading cause of mortality worldwide by 2030 (Kim et al., 2021). Acute exacerbation of COPD (AECOPD) refers to a clinical event characterized by worsening of respiratory symptoms in patients with COPD, leading to changes in symptoms beyond the daily variation range and drug treatment regimens, which is critical for treating COPD disease. This results in reduced quality of life of patients, accelerated decline of lung function, and increased mortality rate of hospitalized patients (Baqdunes et al., 2021; Celli et al., 2021). Presently, there is no effective treatment for AECOPD; therefore, exploring effective prevention and treatment of COPD is one of the most urgent demands of the medical field worldwide.

The respiratory tract constantly exchanges gases with the environment; hence, it is also a system with bacterial colonization. Studies on respiratory microbiota remain in the initial stages. Reportedly, the pulmonary microbiota is closely related to the host’s autoimmune function and participates in the regulation of the immune microenvironment (Cao et al., 2023; Wu et al., 2023). The lungs were presumed to be sterile in healthy individuals; however, owing to the continuous development of medical science and technology, 16S rRNA sequencing has revealed microbial communities detected in the lungs of healthy individuals (Ramsheh et al., 2021; Yagi et al., 2021). The human microbiome includes all forms of microorganisms and their genomes residing within the body of an individual at a specific time, such as in the gut and other mucosal surfaces including the skin, mouth, airways, and vagina (Anand and Mande, 2018, Shi et al., 2021). Ecological imbalance refers to any compositional changes in the microbiome compared with that of healthy individuals (Shi et al., 2021). The low diversity of microbial communities indicates ecological imbalance (Valdes et al., 2018), whereas high diversity is often associated with health and temporal stability (Leitao Filho et al., 2019; Vaughan et al., 2019; Shi et al., 2021).

SJQJD is a medicinal formulation composed of 30 g of *mori cortex*, 15 g of *chrysanthemi indici flos*, 40 g of *semen benincasae*, 20 g of *trichosanthis pericarpium*, 20 g of *pheretima*, 20 g of *fritillariae cirrhosae bulbus*, 50 g of *phragmitis rhizoma*, 150 g of *plantaginis semen*, 20 g of *concretio silicea bambusae*, and 10 g of *glycyrrhizae radix et rhizoma*. SJQJD exerts considerable clinical effects on patients with COPD presenting phlegm-heat obstructing lung (Yee et al., 2022); however, the specific mechanism underlying SJQJD-mediated treatment of COPD remains unclear. Herein, we constructed a COPD rat model and investigated the effects of SJQJD on the pulmonary microbiota of COPD rats through 16S rRNA sequencing. Modern pharmacology indicates that the extract of *mori cortex* has a regulatory effect on oxidative stress (Zhai et al., 2022), *chrysanthemi indici flos, trichosanthis pericarpium, pheretima, fritillariae cirrhosae bulbus, phragmitis rhizoma* all exhibit anti-inflammatory activity (Park et al., 2016; Liu et al., 2020; Tian et al., 2020; Li et al., 2022; Liu et al., 2024), *plantaginis semen* has the function of regulating lipid metabolism and immune response (Sun et al., 2019; Ren et al., 2021), *glycyrrhizae radix et rhizoma* has anti-inflammatory and detoxifying effects (Li et al., 2019; Jiang et al., 2022). These physiological processes are involved in various stages of physiological pathology. However, there is currently limited research by *semen benincaae* and *concretio silicea bambusae*.

## 2 Methods

### 2.1 SJQJD preparation

SJQJD is an internal preparation of Zhongshan Traditional Chinese Medicine Hospital (specific lot number: Guangdong Medicine Preparation Z20071015). All traditional Chinese medicine decoction pieces are provided by the Chinese Pharmacy of Zhongshan Traditional Chinese Medicine Hospital, and identified as qualified authentic products by Deputy Chief Pharmacist He Jianhong. The abovementioned 10 herbs were soaked in water for 30 minutes, and the decoction treatment was performed twice for 1.5 hours. Both decoctions were combined, filtered, concentrated, and added with 200 g of sugar, 3 g of sodium benzoate, and 0.5 g of hydroxyethyl ester. The mixture was boiled and brought to a constant volume of 1 L. Following this, it was allowed to stand for 1 day, and the supernatant was isolated and packaged to complete the preparation.

### 2.2 Animal experiments

Experimental grouping: Specific pathogen free grade 10 week old Wistar male rats (250 ± 20), purchased from Spelford Beijing Biotechnology Co., Ltd. In total, 30 rats were randomly divided into the following five groups (n = 6): control, model, model + SJQJD (high-dose [H]: 1.2 g/mL), model + SJQJD (medium-dose [M]: 0.8 g/mL), and model + SJQJD (low-dose [L]: 0.6 g/mL) groups.

Animal model construction: Both cigarette smoke exposure and lipopolysaccharide (LPS) intratracheal instillation were used to establish the COPD model, as follows: (1) LPS intratracheal instillation: 0.2 mL of LPS solution (1 mg/mL) was instilled into the airway on the 1^st^ and 14^th^ day of modeling; and (2) smoking: from day 2 to 28, rats were transferred to a dedicated disinfection box and exposed to smoke daily (except for day 14), 10 cigarettes per time for 30 minutes, twice a day in the morning and afternoon.

Medication intervention: The control group was not subjected to LPS intratracheal instillation and smoking procedures and was administered 2.5 mL of physiological saline by gavage every day; the model group was administered 2.5 mL of physiological saline by gavage every day; the administration groups were orally administered 2.5 mL of SJQJD (H, M, and L) every day. The alveolar lavage fluids and lung tissues of rats were retrieved after administration for subsequent experiments.

### 2.3 H&E staining experiment

The retrieved lung tissues were fixed with 10% formaldehyde solution. Following this, the tissues were cut into 2-mm thick tissue blocks, which were then dehydrated using gradient ethanol, made transparent using xylene, and embedded in paraffin. Next, the tissue blocks were cut into 5-μm thick slices, stained with H&E, and sealed with neutral gum. The morphology of the lung tissues was observed under a microscope and their photos were captured.

### 2.4 ELISA testing

The alveolar lavage fluids of rats were centrifuged at 4°C at 1800 r/min for 5 minutes, and the supernatants were collected for detection. Next, ELISA was performed to detect interleukin (IL)-6, IL-8, matrix metallopeptidase (MMP)-2, MMP-3, secretory immunoglobulin (sIg)A, and tumor necrosis factor (TNF)-α in the supernatant of the alveolar lavage fluid according to the instructions of the kit (JiangLai, China).

### 2.5 16S rRNA sequencing and bioinformatics analyses

The 16s rRNA sequencing was performed by Shenzhen Weikemeng Technology Group Co., Ltd. using the experimental alveolar lavage fluid, including DNA extraction, polymerase chain reaction-mediated amplification, and Illumina high-throughput sequencing. Bioinformatics analyses were performed using the Wekemo Bioincloud (https://www.bioincloud.tech). Operational taxonomic units (OTUs) were clustered with 97% consistency, and the sequences of OTUs were annotated with species to obtain the corresponding species information and species-based abundance distribution. Additionally, α-diversity analysis was performed utilizing the following evaluation indexes: Chao1 index for evaluating microbial abundance, and Shannon and Simpson indexes for evaluating microbial evenness and abundance. Furthermore, β-diversity was analyzed to compare the diversity among different ecosystems, and cluster analysis was performed on the sample distance matrix to construct a hierarchical visualization of differences among samples. Linear discriminant analysis effect size (LEfSe) analysis was performed to test the significance of differences in species composition and community structure of the grouped samples, further analyzing the microbiome composition of the two groups at the phylum and genus levels, and determining the species abundances with significant differences. Finally, the characteristic microbial communities of each group were screened using machine-learning methods.

### 2.6 Statistic analyses

The GraphPad Prism 9.0 software was used to process data and visualize the data. The comparison between two groups was conducted using t-test method, the comparison between three groups was conducted using one-way ANOVA test method, and the comparison between three groups that did not follow a normal distribution was conducted using Kruskal Wallis test. The *P*-value of < 0.05 was considered statistically significant.

## 3 Results

### 3.1 SJQJD improves the lung tissue morphology in COPD rats

**Figure 1:**
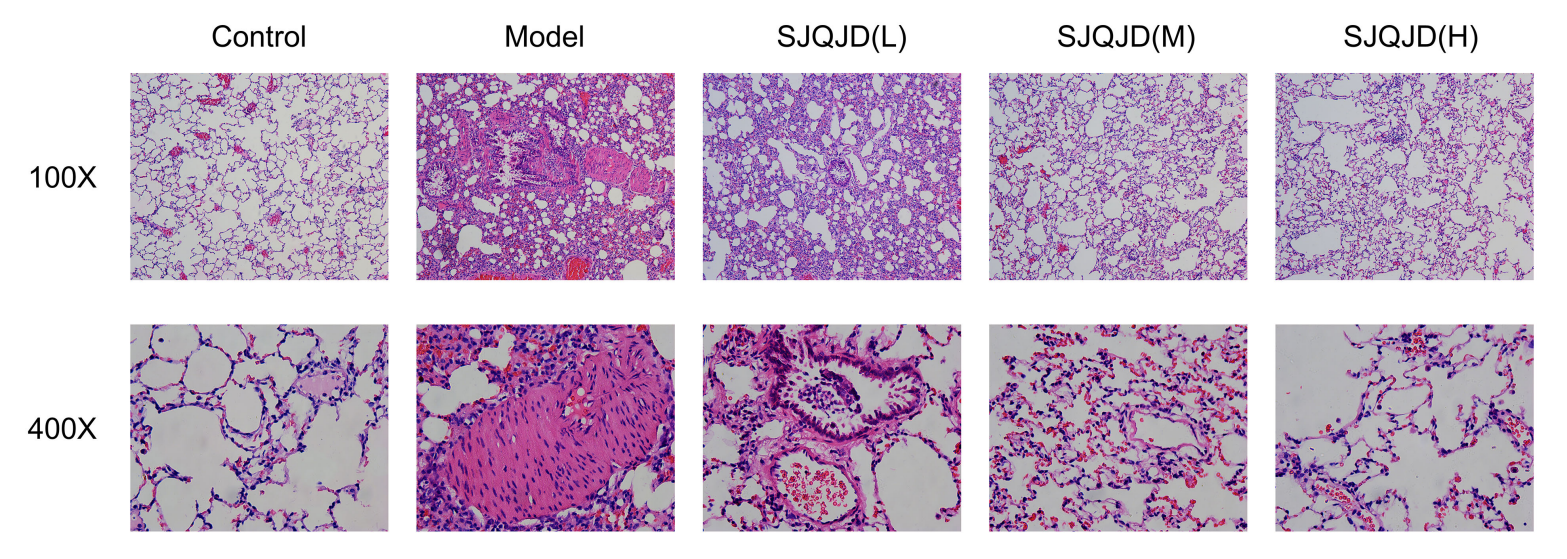
SJQJD improved COPD in rats.

The tissue morphology of the control group was intact with no notable inflammatory cell infiltration (**Figure 1**); however, that of the COPD model group was disordered, with considerable detectable inflammatory cell infiltration and epithelial goblet cell proliferation, indicating the successful establishment of the model. In the SJQJD-H group of rats, a few inflammatory cells were observed in the lung tissue, along with an enlargement of the alveolar spaces. The SJQJD-M group of rats showed reduced aggregation of inflammatory cells in the lung tissue, with some increase in the alveolar septa and inflammatory cell infiltration. The SJQJD-L group of rats demonstrated moderate interstitial inflammatory changes in the lung tissue, along with the widening of the alveolar septa and inflammatory cell infiltration.

### 3.2 SJQJD inhibited the release of inflammatory factors in COPD rats

**Figure 2:**
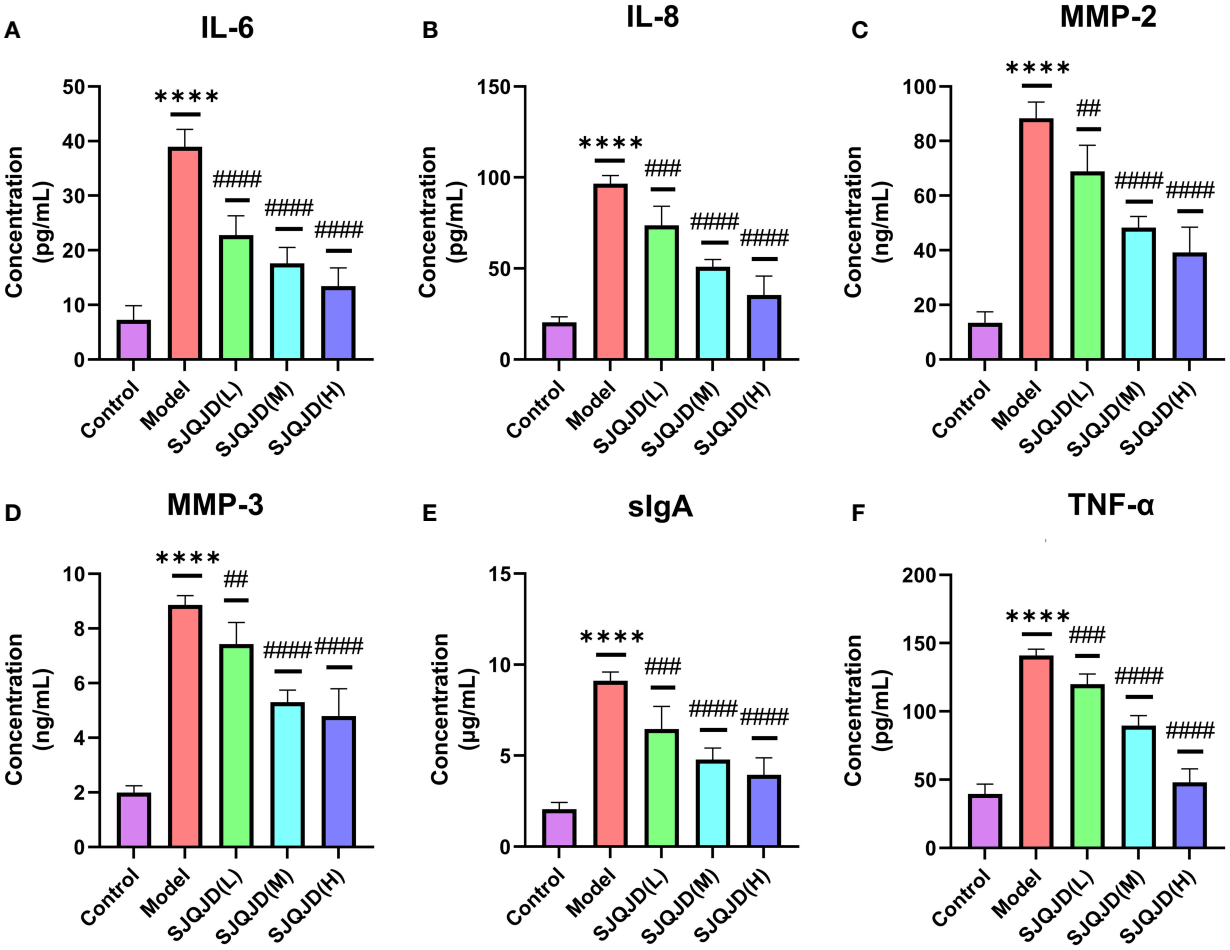
SJQJD improved pulmonary inflammatory response. The level of **(A)** IL-6, **(B)** IL-8, **(C)** MMP-2, **(D)** MMP-3, **(E)** sIgA, and **(F)** TNF- α in rat alveolar lavage fluid. The data was displayed as the mean ± SD (n=6). # *P* < 0.05, ## *P* < 0.01, ### *P* < 0.001, #### *P* < 0.0001, * *P* < 0.05, ** *P* < 0.01, *** *P* < 0.001, **** *P* < 0.0001.

ELISA was performed to detect the content of inflammatory factors in the alveolar lavage fluid. The results showed that compared with the control group, the levels of IL-6 (*P* < 0.0001), IL-8 (*P* < 0.0001), MMP-2 (*P* < 0.0001), MMP-3 (*P* < 0.0001), sIgA (*P* < 0.0001), and TNF-α (*P* < 0.0001) in the model group increased considerably, whereas SJQJD (H, M, and L) suppressed their increase in a concentration-dependent manner (**Figure 2**).

### 3.3 Analysis of the effects of SJQJD on the pulmonary microbiota

**Figure 3:**
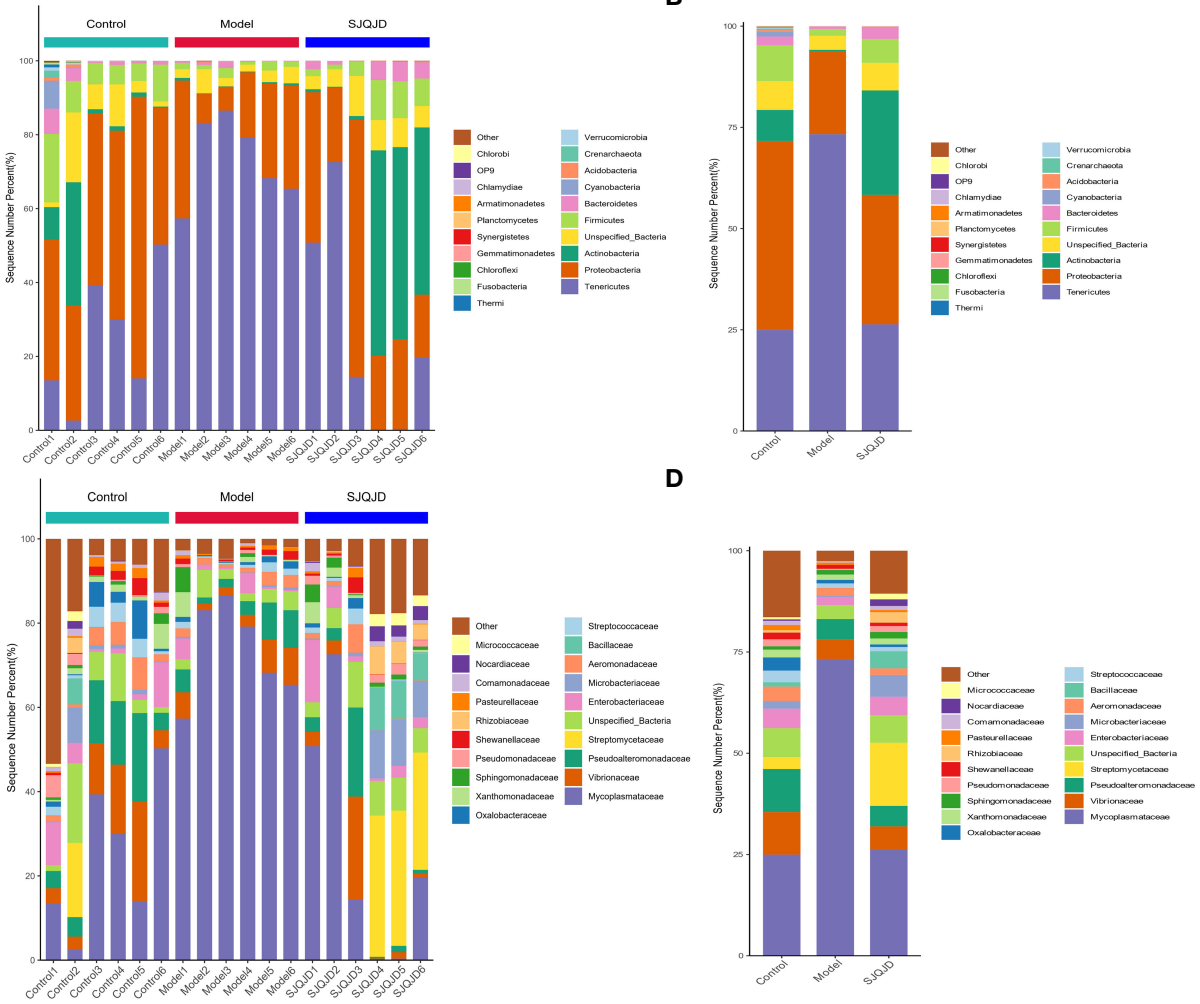
Stacking diagram of the relative abundance of microbial communities. **(A)** The relative abundance of microbial communities at the phylum level in each sample. **(B)** The relative abundance of microbial communities at the phylum level in each group. **(C)** The relative abundance of microbial communities at the genus level in each sample. **(D)** The relative abundance of microbial communities at the genus level in each group.

The composition structures of the lung microbiota of each group of rats were analyzed at the phylum and genus levels through 16S RNA high-throughput sequencing to explore the effects of SJQJD. At the phylum level, the composition of *Tenericutes* notably increased in the model group and considerably decreased in the control and SJQJD groups. In contrast, the compositions of *Proteobacteria, Actinobacteria, Unspecified_Bacteria*, and *Firmicutes* considerably decreased in the model group and markedly increased in the control and SJQJD groups (**Figures 3A, B**). At the genus level, the composition of *Mycoplasmataceae* considerably increased in the model group and markedly decreased in the control and SJQJD groups. In contrast, the composition of *Streptomycetaceae, Enterobacteriaceae, Microbacteriaceae*, and *Bacillaceae* markedly decreased in the model group and notably increased in the control and SJQJD groups (**Figures 3C, D**).

### 3.4 Analyses of α- and β-diversities

**Figure 4:**
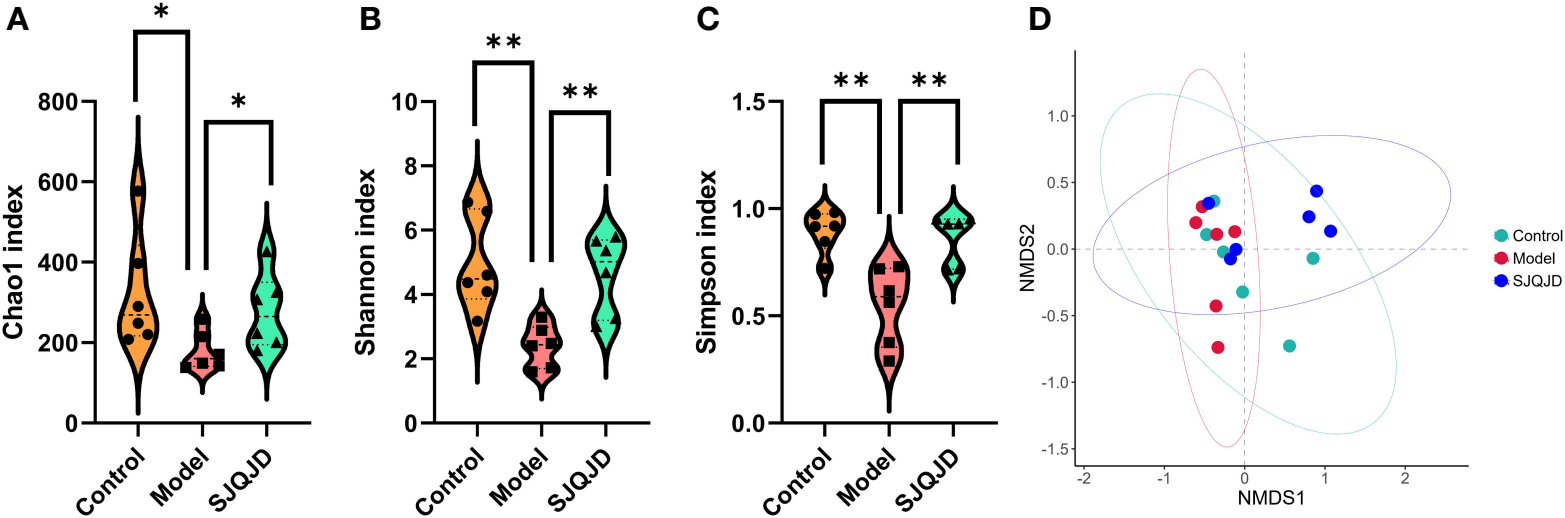
SJQJD improved the diversity of lung microbiota. **(A)** Chao1 index. **(B)** Shannon index. **(C)** Simpson index. **(D)** NMDS. * *P* < 0.05, ** *P* < 0.01.

Compared with the control rats, the α-diversity indexes, namely Chao1 (*P* = 0.0397), Shannon (*P* = 0.0030), and Simpson (*P* = 0.0021) indexes, of COPD rats were markedly reduced (**Figures 4A–C**), suggesting a decrease in both microbial abundance and diversity under COPD conditions. SJQJD reversed the decrease in the aforementioned three indexes, demonstrating its therapeutic effects on COPD rats. β-diversity distance measurements, performed to study the structural changes of the pulmonary microbiota among samples, showed notable differences in the microbial communities of control and COPD rats (**Figure 4D**). However, the SJQJD administration reversed this phenomenon.

### 3.5 Analysis of dominant microbial communities in each group

**Figure 5:**
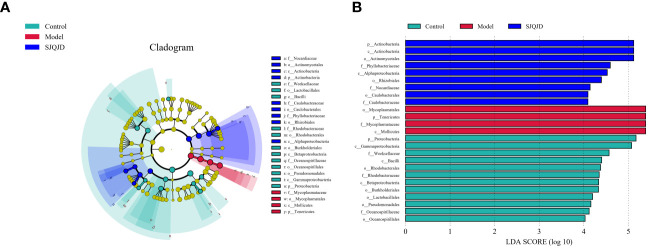
Analysis results of the dominant microbial communities in each group. **(A)** The branch diagram was obtained through LEfSe analysis. **(B)** LDA effect size analysis of major biomarker taxonomic groups.

The bacterial taxa with statistically significant differences among the groups were identified based on the Linear Discriminant Analysis (LDA) value, and the results were visualized by creating a LefSe cladogram (**Figure 5A**) and a histogram of LDA values (**Figure 5B**). *Actinobacteria, Phyllobacteriaceae*, and *Alphaproteobacteria* were the dominant bacterial taxa in the control group, whereas *Mycoplasmatales, Mycoplasmataceae, Tenericutes*, and *Mollicutes* were the dominant taxa in the model group. *Proteobacteria, Gammaproteobacteria, Weeksellaceae*, and others were characteristic taxa for the SJQJD group.

### 3.6 Analysis of species differences in the pulmonary microbiota

**Figure 6:**
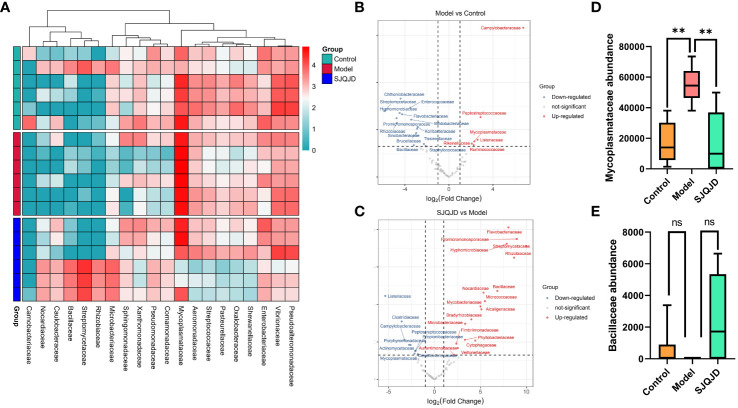
Analysis results of species differences in pulmonary microbiota. **(A)** Heatmap of differential microbial communities among groups. **(B)** Volcano diagram of Model vs. Control. **(C)** Volcano diagram of SJQJD vs. Model. **(D)** The abundance of *Mycoplasmataceae* in three groups. **(E)** The abundance of *Bacillaceae* in the three groups. ** *P* < 0.01, ns, no significant.

The differences in bacterial communities among groups were analyzed at different levels, such as the family level. The results suggest that compared with the control group, the composition of families *Peptostreptococcaceae, Mycoplasmataceae, Rikenellaceae, Listeriaceae*, and *Ruminococcaceae* considerably increased in the model group, whereas that of *Chthoniobacteraceae, Streptomycetaceae*, and *Enterococcaceae* markedly reduced (**Figures 6A, B**). Compared with the model group, the composition of families such as *Bacillaceae, Nocardiaceae*, and *Micrococcaceae* notably increased in the SJQJD group, whereas that of *Listeriaceae, Clostridiaceae, Campylobacteraceae*, and *Mycoplasmataceae* considerably reduced (**Figures 6A, C**). Notably, the abundance of the family *Mycoplasmataceae* (*P* = 0.0021) was considerably increased in the model group, whereas it remained low in both the control and SJQJD groups, suggesting its potential as a therapeutic marker (**Figure 6D**). Although there were no statistically significant differences in *Bacillaceae* abundance among the groups, we observed that this family was almost absent in the model group but exhibited high abundances in both the control and SJQJD groups, suggesting its possible protective role in the disease (**Figure 6E**).

### 3.7 Machine-learning method-based screening of biomarkers

**Figure 7:**
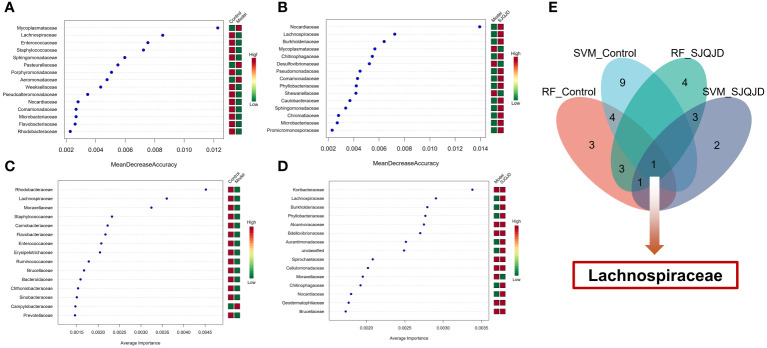
Machine learning screening of microbial biomarkers. **(A, B)** The results of RF method screening for characteristic microbial communities. **(C, D)** The results of SVM method screening for characteristic microbial communities. **(E)** Intersection results of feature microbial communities selected by machine learning.

The markers in the microbiota of each group were further screened through machine learning. The results show that characteristic microbes of the control group obtained using the random forest algorithm included *Lachnospiraceae, Enterococcaceae*, and *Staphylococcaceae*, whereas the model group featured *Mycoplasmataceae, Pasteurellaceae*, and *Aeromonadaceae*. The characteristic microbes of the SJQJD group included Nocardiaceae, Lachnospiraceae, and *Burkholderiaceae* (**Figures 7A, B**; **Table 1**). Additionally, the characteristic microbes of the control group obtained using the support vector machine algorithm included *Rhodobacteraceae, Lachnospiraceae*, and *Moraxellaceae*; those of the model group included Campylobacteraceae and *Moraxellaceae*, and those of the SJQJD group included *Lachnospiraceae, Burkholderiaceae*, and *Phyllobacteriaceae* (**Figures 7C, D**; **Table 1**). By intersecting the results, we found the family *Lachnospiraceae* to be a common marker between the control and SJQJD groups (**Figure 7E**).

**Table 1:** Machine learning screening of characteristic microbial communities in each group.

Group	RF	SVM
Control	*Lachnospiraceae* *Enterococcaceae* *Staphylococcaceae* *Sphingomonadaceae* *Porphyromonadaceae* *Weeksellaceae* *Pseudoalteromonadaceae* *Nocardiaceae* *Comamonadaceae* *Microbacteriaceae* *Flavobacteriaceae* *Rhodobacteraceae*	*Rhodobacteraceae* *Lachnospiraceae* *Moraxellaceae* *Staphylococcaceae* *Carnobacteriaceae* *Flavobacteriaceae* *Enterococcaceae* *Erysipelotrichaceae* *Ruminococcaceae* *Brucellaceae* *Bacteroidaceae* *Chthoniobacteraceae* *Sinobacteraceae* *Prevotellaceae*
Model	*Mycoplasmataceae* *Pasteurellaceae* *Aeromonadaceae* *Desulfovibrionaceae* *Shewanellaceae*	*Campylobacteraceae* *Moraxellaceae*
SJQJD	*Nocardiaceae* *Lachnospiraceae* *Burkholderiaceae* *Chitinophagaceae* *Pseudomonadaceae* *Comamonadaceae* *Phyllobacteriaceae* *Caulobacteraceae* *Sphingomonadaceae* *Chromatiaceae* *Microbacteriaceae* *Promicromonosporaceae*	*Lachnospiraceae* *Burkholderiaceae* *Phyllobacteriaceae* *Aurantimonadaceae* *unclassified* *Chitinophagaceae* *Nocardiaceae*

## 4 Discussion

COPD is a chronic inflammatory disease characterized by persistent restriction of the small airways, and it often affects multiple systems. Studies predict that because of the increasing number of smokers and population aging, the annual COPD-associated mortality and number of patients may exceed 5.4 million (GBD 2017 Causes of Death Collaborators, 2018) by the 2060s. Acute exacerbation, leading to frequent medical visits, hospitalizations, and changes in medication regimens, is a major cause of mortality in patients with COPD (Wu et al., 2014; Vogelmeier et al., 2017; Hua et al., 2020; Li et al., 2021). Microbial cultures indicate that the lung microbiota is related to COPD pathogenesis (Shi et al., 2021), and the advances in metagenomic technologies have further validated this conclusion (Karakasidis et al., 2023).

SJQJD is a hospital-prepared medication formulation reviewed by the drug regulatory authority. Clinical studies have shown the good therapeutic effects of SJQJD when used in combination with Western medicine to treat phlegm-heat obstructed lung-type community-acquired pneumonia. Reportedly, the combined treatment of SJQJD with Western medicine for bronchiectasis can effectively improve the lung function, forced vital capacity (FVC), forced expiratory volume in the 1^st^ second (FEV1), and FEV1/FVC levels of the patients, with a total effective rate of 97.50%, compared with 85.00% in the control group (*P* < 0.05) (Dong et al., 2018). Additionally, clinical research on patients with phlegm-heat congested lung-type AECOPD has shown (Huang et al., 2021) that after treatment with SJQJD in combination with Western medicine, the T lymphocyte subgroup cluster of differentiation (CD)4^+^ and the CD4^+^/CD8^+^ ratio increased, compared with those before the treatment, and CD8^+^ reduced, indicating the significantly better optimization in the combined treatment group than that in the Western medicine control group (*P* < 0.05). This suggested that SJQJD might improve the immune function of patients, thereby enhancing their resistance. These findings imply that SJQJD may exert its therapeutic effect on COPD by altering the lung microenvironment and consequently modulating the lung microbiota.

Herein, a COPD rat model was constructed through a combined approach of cigarette smoke exposure and intratracheal LPS instillation, which is a widely used model for COPD. The COPD model group showed structural disorder with considerable inflammatory cell infiltration and epithelial goblet cell proliferation. However, the SJQJD administration reversed these phenomena, indicating its interventional effects on COPD. Moreover, SJQJD improved pulmonary inflammation.

The lung microbiome plays an important role in maintaining stability within the lungs. The airways of patients with COPD often harbor *Haemophilus influenzae*, *Streptococcus pneumoniae*, and *Moraxella catarrhalis*, which in severe cases can be colonized by *Klebsiella pneumoniae*, *Pseudomonas aeruginosa*, and other *Gram-negative bacteria*. Various factors affect the composition of the respiratory microbiota, including the anatomy of the airways, gender, age, and the immune function of the host (Whiteside et al., 2021). In healthy individuals, the lung microbiota is transient and can be regulated by normal lung defense mechanisms, such as bronchial epithelial cilia movements, coughing, and the immune function of the host. Under healthy conditions, the regional growth conditions generally do not support the extensive proliferation of bacteria, resulting in relatively fewer bacteria. However, inflammatory responses increase the vascular permeability of the airways, providing abundant nutrients, such as amino acids, vitamins, carbon sources, and iron, for bacterial reproduction. Inflammation damages epithelial cells, exposing the basement membrane matrix and promoting bacterial adhesion. Similar to the gut microbiota, dysbiosis of the lung microbiota promotes the persistent progression of COPD (Bowerman et al., 2020). Reduced microbial diversity has been associated with COPD exacerbation events (Sze et al., 2012; Wang et al., 2019; Enaud et al., 2020; Su et al., 2022). Herein, the results showed that the microbial abundance and diversity in the COPD model group were significantly reduced, and SJQJD could considerably reverse this phenomenon, suggesting its role in improving the lung microbiota.

The family *Mycoplasmataceae* includes prokaryotic bacteria such as *Mycoplasma* and *genital Ureaplasma* that are pathogenic to humans (Wood et al., 2021). Reportedly, *mycoplasmas* are one of the common pathogens in patients with COPD (14%) (Lieberman et al., 2001, 2002). *Mycoplasmas* can evade the host immune system, induce apoptosis, generate free radicals, and cause oxidative-reductive imbalance in the cellular glutathione potential through pro-inflammatory cytokines, thus leading to AECOPD (Sessa et al., 2009; Papaetis et al., 2010). The family *Bacillaceae* includes *rod-shaped, endospore-forming, Gram-positive bacteria* (Liu et al., 2015). They are widely found in nature, including both pathogenic and beneficial strains (Hathout et al., 2000). However, their specific role in COPD remains unelucidated. Reportedly, the increase of *Bacillus* in the mouse lungs can aggravate local inflammatory response, resulting in more severe pulmonary emphysema (Richmond et al., 2018), which suggests that *Bacillus* may be a risk factor for COPD. Moreover, a considerably higher population of *Bacillaceae* has been reported in the rat lungs treated with particulate matter 2.5, a COPD-inducing factor, compared with that in the control (Laiman et al., 2023). Furthermore, lower levels of *Bacillus* have been detected in the sputum of patients with COPD (Simpson et al., 2016), indicating that *Bacillus* may play a protective role in COPD. The results of this study indicated a low abundance of *Bacillaceae* in the COPD model, whereas it was considerably higher in the control and SJQJD treatment groups, suggesting that *Bacillaceae* may have a role in combating COPD. Reportedly, most members of the *Lachnospiraceae* family in the gut are associated with decreased lung function; however, the abundances of some members are markedly reduced in COPD (Bowerman et al., 2020; Chiu et al., 2021). To date, only a few studies are on the distribution and role of *Lachnospiraceae* in the lungs. Herein, we discovered *Lachnospiraceae* to be a marker of the microbiota, with a high abundance in the control and SJQJD groups and a low abundance in the model group, suggesting the regulatory role of its members in COPD in the lungs. *Lachnospiraceae* members can metabolize dietary fiber into short-chain fatty acids (SCFAs), and the SCFA levels are positively correlated with the severity of COPD because of their participation in the maturation process of immune cells, which then exert local and systemic anti-inflammatory effects (Jang et al., 2020; Song et al., 2023). The above evidence suggests that *Lachnospiraceae* likely regulate the pulmonary microenvironment and local immune function through their metabolic products.

Furthermore, our study only presented the above results at the animal level. The identification of biomarkers still needs further validation in clinical human specimens. In addition, this article also has certain limitations, as it only observed changes in lung microbiota and did not further verify whether these changes will be involved in the occurrence and development of COPD, as well as the specific mechanisms involved in the process. These are the topics that we need to delve deeper into in the future.

## 5 Conclusion

The findings of this study show that SJQJD can improve COPD in rats. Pulmonary microbiome analysis combined with machine learning identified *Mycoplasmataceae, Bacillaceae*, and *Lachnospiraceae* as potential key biomarkers for SJQJD intervention in COPD; however, more in-depth studies are required to elucidate their specific mechanisms and clinical significance.

## Data availability statement

The 16S sequencing data involved in this article has been uploaded to the figshare database, which can be obtained through the link: https://figshare.com/articles/dataset/16s_sequencing_data_of_Sangju_Qingjie_Detection_SJQJD_on_pulmonary_microbiota_in_COPD_rats/25156670.

## Ethics statement

The animal study was approved by Experimental Animal Ethics Committee of Nankai Hospital in Tianjin. The study was conducted in accordance with the local legislation and institutional requirements.

## Author contributions

ZL: Conceptualization, Data curation, Visualization, Writing – original draft. YH: Conceptualization, Data curation, Project administration, Writing – review & editing. CH: Writing – original draft. XL: Writing – review & editing.
